# A Japanese encephalitis virus biological clone with an E gene point mutation exhibits in vitro and in vivo attenuation of neurovirulence

**DOI:** 10.1099/jgv.0.002137

**Published:** 2025-09-01

**Authors:** Shu Pin Yu, Kien Chai Ong, Soon Hao Tan, Tomohiro Ishikawa, David Perera, Yuan Teng Hooi, Kum Thong Wong

**Affiliations:** 1 Department of Pathology, Faculty of Medicine, Universiti Malaya, Kuala Lumpur, Malaysia; 2 Department of Biomedical Science, Faculty of Medicine, Universiti Malaya, Kuala Lumpur, Malaysia; 3 Department of Microbiology, Dokkyo Medical University School of Medicine, Tochigi, Japan; 4 Institute of Health and Community Medicine, University Malaysia Sarawak, Sarawak, Malaysia; 5 Infection and Immunity Research Strength, Jeffrey Cheah School of Medicine and Health Sciences, Monash University Malaysia, Selangor, Malaysia; 6 Jeffrey Cheah School of Medicine and Health Sciences, Monash University Malaysia, Selangor, Malaysia

**Keywords:** biological clones, E protein, *Japanese encephalitis *virus, neurovirulence, neuroinvasion, Y59H

## Abstract

The Japanese encephalitis virus (JEV), a leading cause of viral encephalitis, exists as similar but non-identical biological clones whose genomic variations/mutations may determine neurovirulence. Two biological clones purified from a brain-derived, clinical isolate were tested *in vitro* for neurovirulence using human neuronal cells (SK-N-MC) and mouse neuronal cells (NIE-115) and *in vivo* on a footpad-inoculation mouse model. One clone (JEV-M) demonstrated significantly reduced infectivity in both neuronal cells and the mouse model compared to another clone (JEV-V). Of the 2 *E* gene point mutations in JEV-M, only the T175C mutation, which translates as an E protein residue 59, amino acid tyrosine to histidine change (Y59H), was found to be the neurovirulence determinant as confirmed by testing with infectious clones with or without these mutations. These novel findings could further our understanding of JEV neuropathogenesis and may be useful for future vaccine development.

## Introduction

Japanese encephalitis virus (JEV) is an enveloped, single-stranded positive-sense RNA arbovirus belonging to the family *Flaviviridae* and genus *Orthoflavivirus*. The ~11 kb genome comprises capsid, pre-membrane and envelope (*E*) genes that encode for three corresponding structural proteins and seven non-structural genes: *NS1*, *NS2A* and *B*, *NS3*, *NS4A* and *B* and *NS5*. The ORF is flanked by 5′ and 3′ untranslated regions [1,3].

JEV is one of the leading causes of mosquito-borne viral encephalitides with an annual global estimation of 68,000 Japanese encephalitis (JE) cases and 13,600 to 20,400 deaths in affected areas [4]. In endemic areas, JE largely involves children, but in non-endemic areas, all age groups are at risk of infection [1]. With a 1:25 to 1:1000 symptomatic to asymptomatic ratio [1,5], fatality rate ranges from 25 to 50%, and more than 50% of the survivors suffer permanent neurological sequelae [6].

Like other orthoflaviviruses, including tick-borne encephalitis virus (TBVE) and West Nile virus, JEV replicates in the absence of proof-reading and repair of the newly synthesized viral RNA. Hence, the virus exists as a mixture of closely similar genotypes, termed biological clones [7,8]. Viral biological clones may result in biological differences, including differences in cell tropism, virulence and host range, and resistance to antiviral agents and host immune responses [7,9]. In the laboratory, a highly virulent strain of TBVE was repeatedly cultured in mammalian porcine kidney cells, resulting in the production of three viral biological clones. These biological clones showed variations in plaque sizes and virulence when tested in a mouse model [10]. Relatively little is known about genomic diversities in JEV biological clones and how they may impact neurovirulence.

In this study, we purified biological clones derived from a clinical isolate to investigate viral genomic differences that could determine neurovirulence. Following our *in vitro* results that suggested that *E* gene mutations may impact neurovirulence, we also tested the biological clones and corresponding infectious clones (IC) on a footpad-inoculation JE mouse model that we had previously shown to be a good *in vivo* neurovirulence model [11]. Our *in vitro* and *in vivo* results suggest that *E* gene mutations in JEV may play a significant role in neuropathogenesis.

## Methods

### Cell lines

Human neuroepithelioma cells (SK-N-MC) (ATCC-HTB-10, USA) and mouse neuroblastoma cells (NIE-115) (ATCC-CRL-2263, USA) were cultured in Dulbecco’s Modified Eagle Medium (Sigma-Aldrich, USA), supplemented with 10% fetal bovine serum (FBS) (Hyclone, Fisher Scientific, USA) and gentamicin. *Aedes albopictus* cells (C6/36) (ATCC-CRL-1660, USA) were cultured in Roswell Park Memorial Institute medium (RPMI-160) (Sigma-Aldrich, USA), supplemented with 10% FBS and gentamicin.

### Biological clone isolation and purification

A JEV clinical isolate (CNS138/9), originating from the brain of a deceased patient in Sarawak, Malaysia [12], was purified using a limiting dilution method as previously described [13]. In brief, C6/36 cells seeded in 96-well plates (1.5×10^4^ cells/well) were infected with 10-fold serial dilutions of CNS138/9 virus stock in quadruplicate sets. At 7 days post-infection (dpi), supernatant was collected from each well. Wells with the highest dilutions and viral antigen-positive cells underwent one more round of limiting dilution purification as before. As proof of infection, cells were stained by immunocytochemistry (ICC) for viral antigens because infected C6/36 cells do not show obvious cytopathic effects (CPE). Cells were methanol-fixed, followed by application of anti-JEV primary antibody, Jath-160 (obtained from Dr. Takasaki, National Institute of Infectious Diseases, Japan), at 1:5000 dilution, 4 °C, and overnight incubation. This was followed by 30 min ENVISON HRP-conjugated goat anti-mouse secondary antibody (Dako, Denmark) at room temperature (RT). Chromogen development was done using 3,3′-diaminobenzidine at a 1:50 dilution (Dako, Denmark). All purified biological clones obtained were propagated once in C6/36 cells and virus-titrated using the CCID_50_ assay.

### Virus titration by CCID_50_ assay

The CCID_50_ assay was performed with slight modifications as previously described [14]. In brief, C6/36 cells seeded in 96-well plates (1.5×10^4^ cells/well) were infected with 10-fold serial dilutions of JEV in quadruplicates. Infected cells were methanol-fixed and immunohistochemistry (IHC)-stained for viral antigens as a replacement for CPE observation, as described above. Viral titres were calculated using Karber’s method [15].

### Biological clone infection of SK-N-MC and N1E-115 cells

SK-N-MC cells seeded into 24-well plates (8×10^4^ cells/well) overnight were infected with the two purified biological clones, JEV-V (GenBank accession no. PV210294) and JEV-M (GenBank accession no. PV210295), at a multiplicity of infection (MOI) of 10. Unbounded viruses were removed by washing with PBS (pH 7.4) after an hour of pre-adsorption. Uninfected controls and infected cells were monitored up to 120 hours post-infection (hpi). At 120 hpi, cells were methanol-fixed and ICC-stained for viral antigens as before. Based on the results that showed progressive CPE, cell detachment and viral antigen positivity as evidence of neurovirulence, JEV-V was classified as neurovirulent, while JEV-M was found to be attenuated.

To further compare the relative neurovirulence between human and mouse neuronal cells, SK-N-MC and N1E-115 cells were seeded into 12-well plates (10^5^ cells/well) for a day and then infected with JEV-V and JEV-M at MOIs of 10, 1 and 0.1. Unbound viruses were removed from cell cultures by PBS after 1 h pre-adsorption. To determine the one-step growth curves of JEV-V and JEV-M in neuronal cells, SK-N-MC and N1E-115 cells were seeded into 12-well plates (10^5^ cells/well) and infected at an MOI of 10. Total cell lysates from three independent sets of experiments were collected at 24, 48, 72 and 96 hpi, freeze-thawed and viral titrated.

To determine whether the reduced infection of JEV-M was specific to neuronal cells, Vero cells, a non-human primate kidney cell line, were used to assess JEV-M infectivity in non-neuronal cells. Briefly, Vero cells seeded into 12-well plates (10^5^ cells/well) for a day were infected with JEV-V and JEV-M at an MOI of 1. Total cell lysates from three independent sets of experiments were collected at 24, 48, 72 and 96 hpi, freeze-thawed and viral titrated.

### Virus binding assay

The binding capacities of JEV-V and JEV-M virions to neuronal cells were assessed using a virus binding assay. SK-N-MC and NIE-115 cells were seeded into 12-well plates (1×10^5^ cells/well) overnight. The cells were pre-chilled at 4 °C for 1 h prior to infection with JEV-V and JEV-M at an MOI of 10 for 2 h at 4 °C. Unbound viruses were removed by washing the cells three times with ice-cold PBS. Uninfected cells served as negative controls. Total RNA was extracted using the RNeasy Plus Mini kit (QIAGEN, Germany), according to the manufacturer’s instructions. Approximately 100 ng RNA was reverse-transcribed to cDNA using the Transcriptor First Strand cDNA Synthesis Kit (Roche, Switzerland), followed by absolute quantification of viral RNA via SYBR green real-time PCR using JEV-specific primers (Table S1, available in the online Supplementary Material).

### Whole genome sequencing and bioinformatics analysis

The whole genomes of JEV-V and JEV-M were sequenced. Viral RNA was extracted and reverse-transcribed using the High Pure Viral RNA Extraction Kit (Roche, Switzerland) and the Transcriptor First Strand cDNA Synthesis Kit (Roche, Switzerland), respectively. For whole-genome sequencing, the cDNAs were PCR-amplified using HotStarTaq DNA Polymerase (QIAGEN, Germany) and JEV-specific primers (Table S1) to amplify overlapping viral genomic cDNA fragments. The PCR conditions followed the manufacturer’s protocol, adjusted for annealing temperatures (T_a_) and extension times for optimization: 95 °C for 15 min, 35 cycles of denaturation, annealing and extension at 94 °C for 1 min, 50–60 °C (depending on primer pairs used) for 1 min, 72 °C for 3–4 min (depending on primer pairs used) and final extension at 72 °C for 10 min. The 3′ and 5′ terminal sequences were obtained using 3′ and 5′ RACE [16], respectively. PCR products were purified using the High Pure PCR Product Purification Kit (Roche, Switzerland) and were sent for Big Dye Terminator Sanger sequencing (Applied Biosystems, Invitrogen, USA). Nucleotide sequences with overlapping reads that covered the amplicons on both strands were assembled using DNA Baser v 4.16.0 (HeradeBioSoft S.R.L, Romania) to obtain the complete genome.

All coding sequences were translated to the corresponding amino acids and aligned using the AlignPlus 4 software (S and E Software, USA). The E protein 3D models were predicted using the web-based protein analysis software I-TASSER [17,19]. Qualities of the predicted models were assessed using a C-score within the range of 0.5 to 2. The structural similarities between the predicted models and the native structures were determined using TM-scores, where scores of more than 0.05 were used as indications of correct topology.

### Generation of infectious clones

ICs were generated using standard molecular cloning methods and subcloning of the PCR-amplified JEV-V genome into the previously established MuarFLC plasmid [20]. In brief, the Not-1 JEV-V 5′ end fragment of around 6.5 kb was PCR-amplified using primer pairs Not-T7-JE-F and 13R (Table S1), and the PCR product was inserted into the MuarFLC plasmid at the Not-1-Spel (Fast Digest FD0593, FD1253, Thermo Scientific, USA) cutting site. The remaining fragment of about 4.5 kb was fused with hepatitis D virus (HDV) ribozyme and SWAI using fusion PCR [21] and primer pairs 24F and HDV-10964R, HDV-F and HDV-R-SWAI (Table S1). The PCR product was inserted at the SpeI-SwaI (Fast Digest FD1253, FD1244, Thermo Scientific, USA) cutting site. Transformation was done using TOP 10 *Escherichia coli-*competent cells (Invitrogen, USA). After confirming that *in vitro*-transcribed RNA from the final product JEV-V_IC_ could produce IHC-confirmed viral antigens by C6/36 cell transfection, the JEV-V_IC_ genome was sequenced to confirm 100% homology with wild type (WT) JEV-V.

Given that *E* gene mutations have been more commonly reported as neurovirulence determinants, our approach prioritized infectious clones reproducing the two *E* gene mutations (T175C and G979A) found in JEV-M but not in JEV-V. To produce the IC (JEV-V_IC/T175C/G979A_) that contained both mutations, the PCR product generated from primer pairs Not-T7-JE-F and 23 R using JEV-M cDNA as the template was inserted into the JEV-V_IC_ plasmid at the Not-1-Xhol cutting site (Fast Digest FD0593, FD0698, Thermo Scientific, USA). The ICs that have a single nucleotide *E* gene mutation (either T175C or G979A; designated as JEV-V_IC/T175C_ and JEV-V_IC/G979A_, respectively) were generated using PCR-based, site-directed mutagenesis with primer pairs containing the intended point mutations and the JEV-V_IC_ plasmid DNA as a template [22]. The *E* gene T175C mutation was created using primer pairs Not-T7-JE-F and fragment 1 mt1-R, fragment 2 mt1-F and 23R, while the G979A mutation was inserted using primer pairs Not-T7-JE-F and fragment 1 mt2-R, fragment 2 mt2-F and 23R (Table S1). After PCR, the products were subcloned into the JEV-V_IC_ plasmid at the Not1-Xhol cutting site (Fast Digest FD0593, FD0698, Thermo Scientific, USA) and confirmed by sequencing. All PCR amplifications were done using the high-fidelity Q5 DNA polymerase (New England BioLabs, Ipswich, MA) following manufacturer’s protocol, but with adjusted T_a_ and extension times for optimal PCR amplification: 98 °C for 30 s, 35 cycles of denaturation, annealing and extension at 98 °C for 10 s, 57–72 °C (depending on primer pairs) for 30 s, 72 °C for 2–7 min (depending on primer pairs) and final extension at 72 °C for 2 min.

In order to recover infectious viral progenies, Swal linearized plasmid IC DNAs were *in vitro* transcribed using the MEGAscript T7 Transcription kit (Ambion, Thermo Fisher Scientific, USA) with the 5′ cap analogue m^7^G(5′)ppp(5′)A (S1405S) (New England Biolabs, Ipswich, USA). The *in vitro*-transcribed RNAs were treated with TURBO DNase1 at 37 °C for 15 min to remove plasmid templates and subsequently purified. Infectious progenies were recovered by transfection of *in vitro-*transcribed viral RNA in C6/36 cells. Virus stocks were propagated once in C6/36 cells and titrated using the CCID_50_ assay as before. The *E* gene sequences of these virus stocks were reconfirmed, using primer pairs targeting partial membrane (M), full envelope (E) and partial non-structural 1 (NS1) gene sequence (Table S1) and all subsequent steps were as described above for whole-genome sequencing.

### Phenotypic characterization of infectious clones’ viral progeny

*In vitro* neurovirulence of JEV-V_IC_, JEV-V_IC/T175C_, JEV-V_IC/G979A_ and JEV-V_IC/T175C/G979A_ viral progenies was tested using SK-N-MC and NIE-115 cells seeded into 12-well plates (10^5^ cells/well) at MOIs of 10, 1 and 0.1, and at 120 hpi, collected supernatants were virus-titrated as before. Similarly, the one-step growth curves in both neuronal cells infected at an MOI of 10 were determined in supernatants collected at 24, 48, 72 and 96 hpi as before.

### Infection of the Japanese encephalitis mouse model by biological and infectious clones

To compare the *in vivo* neurovirulence of JEV-V and JEV-M biological clones, groups of 2-week-old ICR mice (*n*=4 per group) were inoculated via the right hind footpad with 20 µl of 10^6^, 10^5^ and 10^4^ CCID_50_/ml of either JEV-V or JEV-M, respectively. Additionally, four groups of mice (*n*=4 per group) were footpad-inoculated with 20 µl of infectious viral progenies at 10^4^ CCID_50_/ml obtained from JEV-V_IC_, JEV-V_IC/T175C/G979A_, JEV-V_IC/T175C_ and JEV-V_IC/G979A_, respectively. An uninfected control group (*n*=4) was footpad-inoculated with 20 µl PBS.

All animals were monitored twice daily up to 21 dpi. Animals that developed severe signs of infection or became moribund were euthanized via isoflurane inhalation. Animals that remained healthy were euthanized at the end of the observation period using the same method. Kaplan–Meier survival curves were plotted for each group of animals. Animal carcasses were fixed in 10% buffered formalin, routinely processed and paraffin-embedded for histopathological analysis. Paraffin blocks were sectioned at 4 µm thickness, and tissue sections were mounted onto 3-aminopropyltriethoxysilane (Sigma-Aldrich, USA)-coated slides for haematoxylin and eosin staining and IHC to detect viral antigens.

All animal experiments were approved by the Institutional Animal Care and Use Committee, Faculty of Medicine, University of Malaya (ethics number: 170819/PATHO/PS/WKT, 2017-200106/PATHO/R/WKT), and were conducted in the AAALAC International-accredited Animal Experimental Unit.

### Immunohistochemistry for viral antigen detection in animal tissues

IHC was performed using a standard ENVISION immunoperoxidase technique [23,24]. Following a 20-min peroxidase blocking, 30-min heat-mediated antigen retrieval (Tris-EDTA/0.05% Tween 20, pH 9.0) and 20-min serum blocking (1:20 dilution) at RT, tissue sections were incubated overnight at 4 °C with anti-JEV E protein antibody (GTX125876, GeneTex, USA) at a 1:2,500 dilution. Sections were then incubated in ENVISION HRP-conjugated goat anti-rabbit secondary antibody (Dako, Denmark) at a 1:1 dilution for 30 min at RT. Chromogen development was carried out using DAB (Dako, Denmark) at 1:50 dilution, followed by Harris haematoxylin counterstaining. All washing steps between incubations were performed with TBS containing 0.05% Tween 20 (pH 8.0) three times.

### Statistical analysis

Statistical analysis was performed on the means of three independent experiments, expressed as mean±sd. Normally distributed data were analysed with T-tests or ANOVA. Non-parametric analyses were performed for non-normally distributed data. Data normality was determined by the Shapiro–Wilk analysis. Statistical significance was set as *P*≤0.05. All statistical analyses were performed using SPSS software, version 17.0.

## Results

### Reduced JEV-M infectivity in neuronal cells

**Fig. 1.**
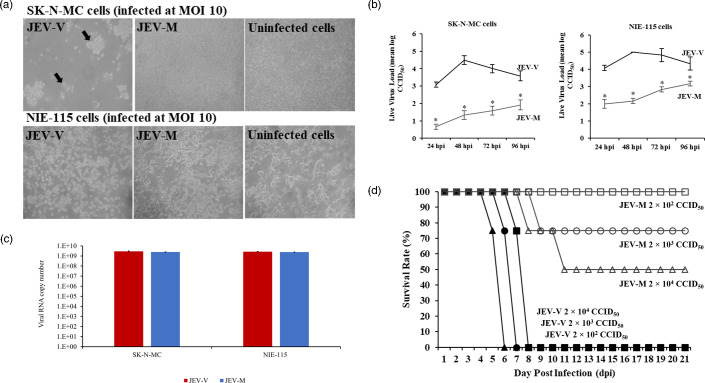
Comparative analysis of WT JEV-V and JEV-M. (**a**) Infection of SK-N-MC and NIE-115 cells with JEV-V and JEV-M. At an MOI of 10, SK-N-MC cells infected with JEV-V displayed CPE, including cell clumping, shrinkage and detachment (indicated by arrows). In contrast, CPE was minimal or absent in JEV-M-infected and uninfected cells. In NIE-115 cells, JEV-V infection at MOI 10 resulted primarily in cell rounding, while CPE was minimal or absent in both JEV-M-infected and uninfected cells (original magnification: 10× objective) (*Note: NIE-115 cells generally do not reach 100% confluency, unlike SK-N-MC cells.*) (**b**) Replication kinetics of JEV-V and JEV-M in SK-N-MC and NIE-115 cells. At an MOI of 10, one-step growth curves showed significantly higher (*P*≤0.05) viral titres in JEV-V-infected cells than in JEV-M-infected cells at all time points. JEV-V replication peaked at 48 hpi and decreased gradually thereafter, while JEV-M replication gradually increased over time. (**c**) Mean viral RNA copy number ±sd bound to SK-N-MC and NIE-115 cells following 2 h of incubation at 4 °C. No significant differences in binding efficiencies of JEV-V and JEV-M were observed (*P*>0.05). The *Y*-axis denotes the amount of virus bound, quantified as viral RNA copy number. (**d**) Survival graphs of mice following footpad inoculation with JEV-V, JEV-M and their respective infectious clones. All mice infected with 20 µl of 10^6^, 10^5^ and 10^4^ CCID_50_/ml JEV-V succumbed to the infection at 6, 7 and 8 dpi, respectively. In contrast, mice infected with the same volume of JEV-M at 10^6^ and 10^5^ CCID_50_/ml showed survival rates of 50% and 75%, respectively, while 100% of mice survived infection with 10^4^ CCID_50_/ml. * indicates statistical significance, *P*-values≤0.05.

At the MOI of 10, JEV-V demonstrated CPE and cell detachment in SK-N-MC and N1E115 cells starting at 48 hpi (Fig. 1a). In contrast, JEV-M showed reduced infectivity in SK-N-MC and N1E115 cells with minimal CPE at 120 hpi (Fig. 1a).

At the MOI of 10, the one-step growth kinetics of JEV-M in SK-N-MC and NIE-115 cells showed modest and gradual increases from 24 to 96 hpi, with the viral titres ranging from mean log 0.5 to 3 CCID_50_ (Fig. 1b). However, JEV-V growth was more rapid, peaking at 48 hpi, and decreased gradually thereafter with viral titre range of mean log 3 to 5 CCID_50_ (Fig. 1b). At all corresponding time points, JEV-M titres were significantly lower (*P*≤0.05) than JEV-V titres (Fig. 1b).

### Binding efficiency of JEV-V and JEV-M in neuronal cells

The binding efficiency of JEV-V and JEV-M virions to neuronal cells was evaluated after 2 h of incubation at 4 °C. In both SK-N-MC and NIE-115 cells, there were no statistically significant differences in the amount of cell-bound JEV-M virions compared to JEV-V (*P*>0.05). In SK-N-MC cells, the mean viral RNA copy number±sd was (2.87±0.32) × 10⁹ for JEV-V and (2.50±0.063) × 10⁹ for JEV-M. Similarly, in NIE-115 cells, the viral RNA copy number was (2.68±0.16) × 10⁹ for JEV-V and (2.43±0.081) × 10⁹ for JEV-M (Fig. 1c).

### Attenuation of JEV-M neurovirulence in the mouse model

Death/moribund/severe infection rates of 50%, 25% and 0% were observed in mice footpad-inoculated with 10^6^, 10^5^ and 10^4^ CCID_50_/ml JEV-M, respectively. Severely infected animals demonstrated signs such as ruffled fur, hunched back posture, paralysis and seizures. These signs in JEV-M-infected animals generally started from a mean 8.5±0.71 dpi in 2 of 4 animals and 8 dpi in 1 of 4 animals for viral doses of 10^6^ and 10^5^ CCID_50_/ml, respectively. At the viral dose of 10^4^ CCID_50_/ml, all the surviving JEV-M-infected animals did not show any signs of infection and appeared healthy throughout the observation period. All JEV-V infected mice had the same signs of infection, which started earlier from mean dpi of 4.25±0.96, 5.5±0.58 and 6.5±0.58, for viral doses 10^6^, 10^5^ and 10^4^ CCID_50_/ml, respectively. Moreover, these mice died or became moribund earlier at a mean dpi of 5.75±0.5, 6.75±0.5 and 7.75±0.5, respectively (Fig. 1d).

**Fig. 2.**
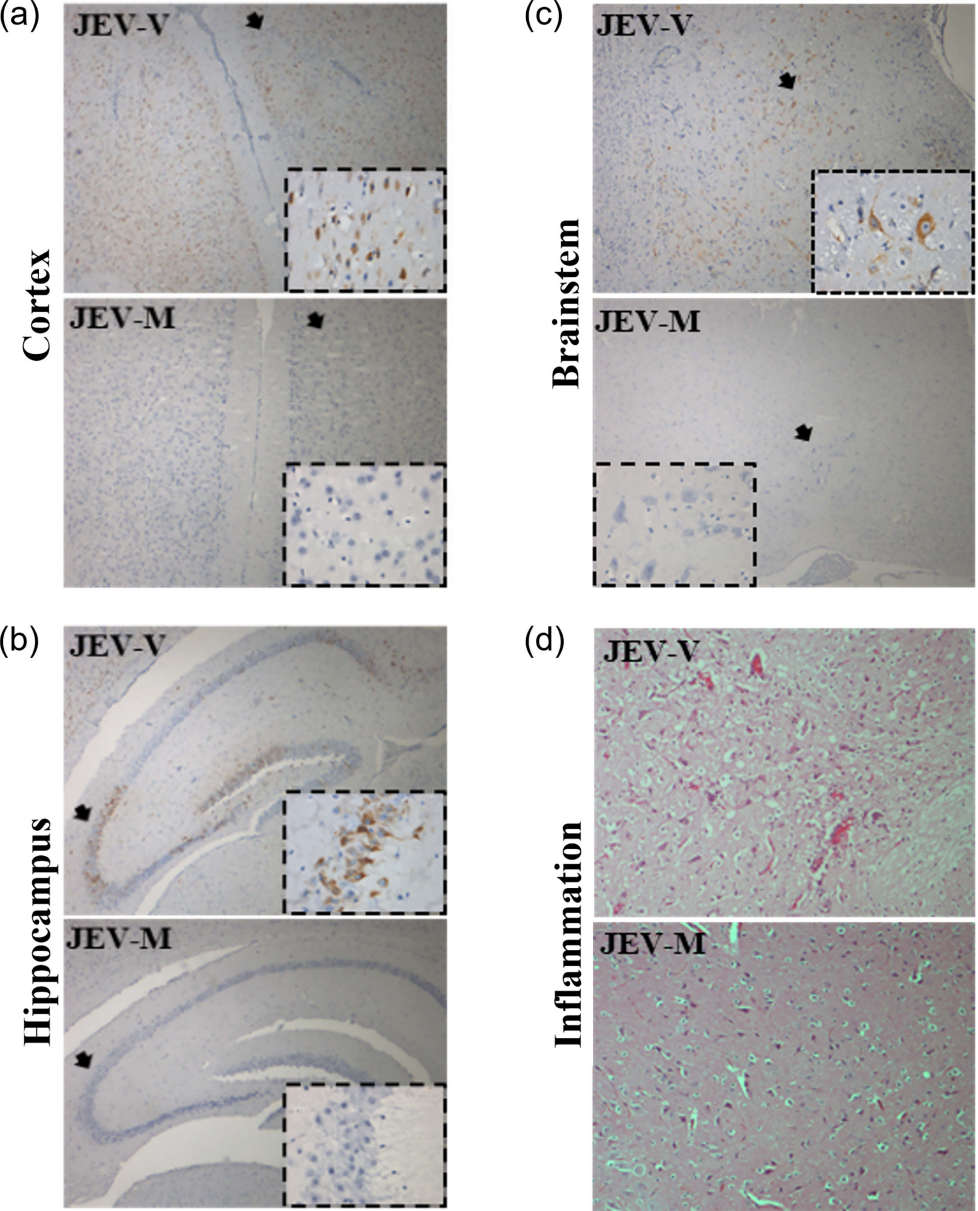
Viral antigen and inflammation in JEV-V- and JEV-M-infected mice. Viral antigen positivity and distribution in comparable and representative sections of the cerebral cortex (**a**), hippocampus (**b**) and brainstem (**c**) in JEV-V-infected mice, but not in JEV-M-infected mice (original magnification: 4× objective; inset: 40× objective). The higher magnification images in the insets, taken from the areas indicated by arrows, either showed neuronal cell body cytoplasmic localization of viral antigens or their absence. (**d**) Inflammation was detected in the brainstem of JEV-V-infected mice, but not in JEV-M-infected mice (original magnification: 10× objective). All animals were footpad-infected with the same dose of 20 µl 10^4^ CCID_50_/ml.

In all the animals infected with 10^4^ CCID_50_/ml JEV-M, there was no inflammation, and viral antigens were not detected in the central nervous system (CNS) of surviving animals euthanized on 21 dpi (*n*=4). However, at this same dose, all JEV-V-infected animals (*n*=4) showed inflammation (Fig. 2d) and extensive viral antigens in the cerebral cortex, thalamus, hippocampus, hypothalamus, brainstem and the spinal cord (Fig. 2a–c) . All uninfected control animals appeared healthy throughout the experimental period, and CNS inflammation and viral antigens were absent.

### Whole genome comparison of JEV-M and JEV-V

Whole genome sequencing showed only four nucleotide differences between JEV-M and JEV-V biological clones. In JEV-M, a thymine to cytosine (T175C) mutation was observed in the *E* gene at position 175, resulting in the tyrosine to histidine (Y59H) amino acid change on the E protein at position 59. A second *E* gene guanine to adenine (G979A) substitution at position 979 resulted in an alanine to threonine (A327T) change on the E protein at position 327 (Table 1). In addition, a cytosine to thymine change in the NS5 gene at position 628 resulted in the proline to serine amino acid change in the NS5 protein at position 210. A nucleotide, cytosine to thymine change in the 3′-UTR at position 10528 was also identified in JEV-M (Table 1).

**Table 1.** Nucleotide and amino acid differences between JEV-M and JEV-V biological clones identified through whole-genome sequencing

Biological clone	Neurovirulence	*E* gene		*E* gene		*NS5* gene		3′UTR
		Nucleotide position (175)	Amino acid position (59)	Nucleotide position (979)	Amino acid position (327)	Nucleotide position (628)	Amino acid position (210)	Nucleotide position (10528)
JEV-M	Attenuated	Cytosine	Histidine	Adenine	Threonine	Thymine	Serine	Thymine
JEV-V	Neurovirulent	Thymine	Tyrosine	Guanine	Alanine	Cytosine	Proline	Cytosine

E, Envelope; NS5, non-structural protein 5; 3'UTR, 3' untranslated region.

**Fig. 3.**
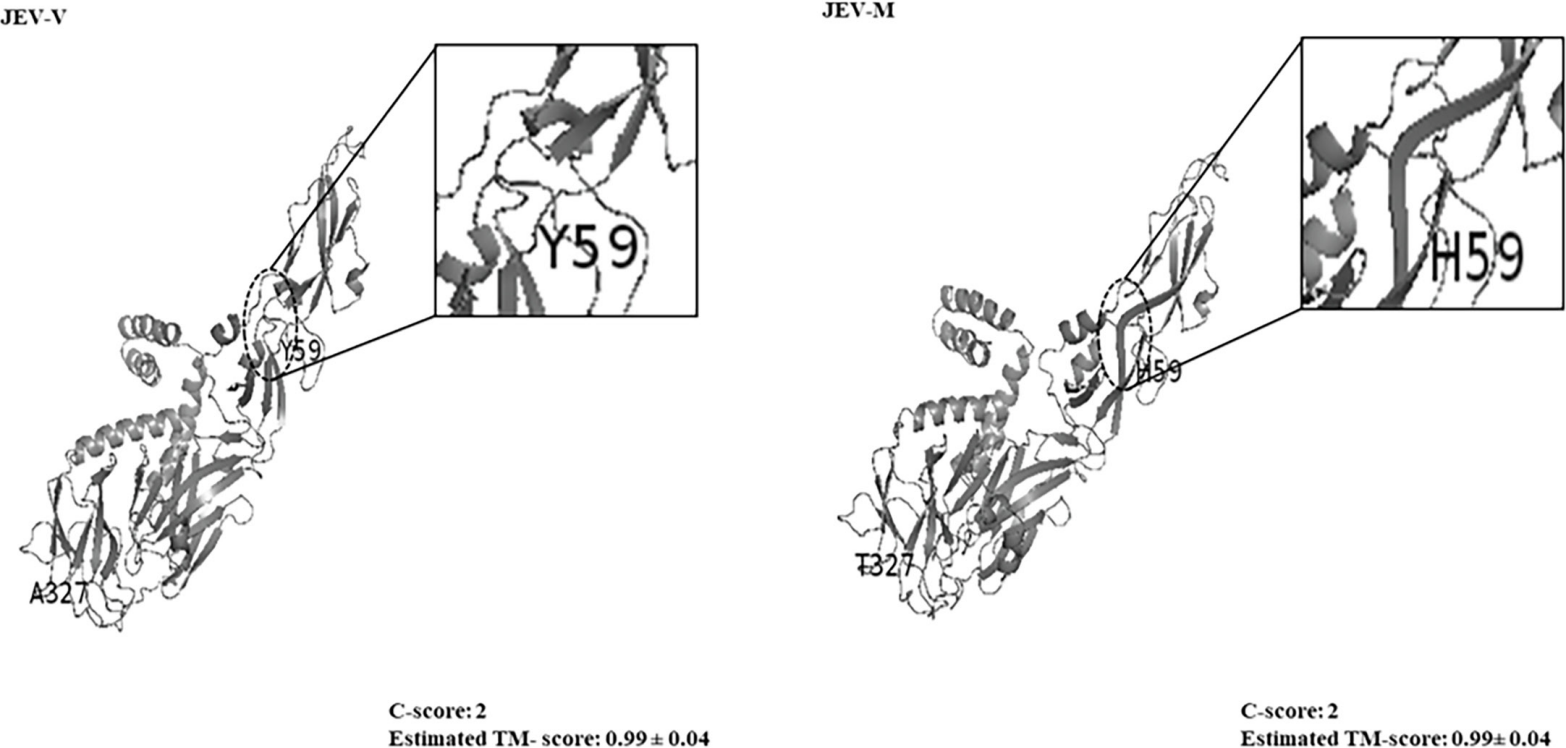
Predicted 3D models of E proteins of JEV-V and JEV-M. The Y59H amino acid substitution in JEV-M resulted in a secondary structure change involving residues 59 to 62 (circle; arrow) from *α*-helixes to *β*-strands. The C-scores and the estimated TM-scores for both models were 2 and 0.99±0.04, indicating models of high confidence and correct topology.

The Y59H amino acid change in JEV-M, located on the E protein domain II, resulted in secondary structure changes from alpha helices to *β*-strands from residues 59 to 62. On the other hand, the domain III, A327T amino acid substitution did not appear to result in any change to the *β*-strand structure on residue 327 (Fig. 3).

### Infectious clone with T175C mutation showed a similar phenotype to JEV-M

**Fig. 4.**
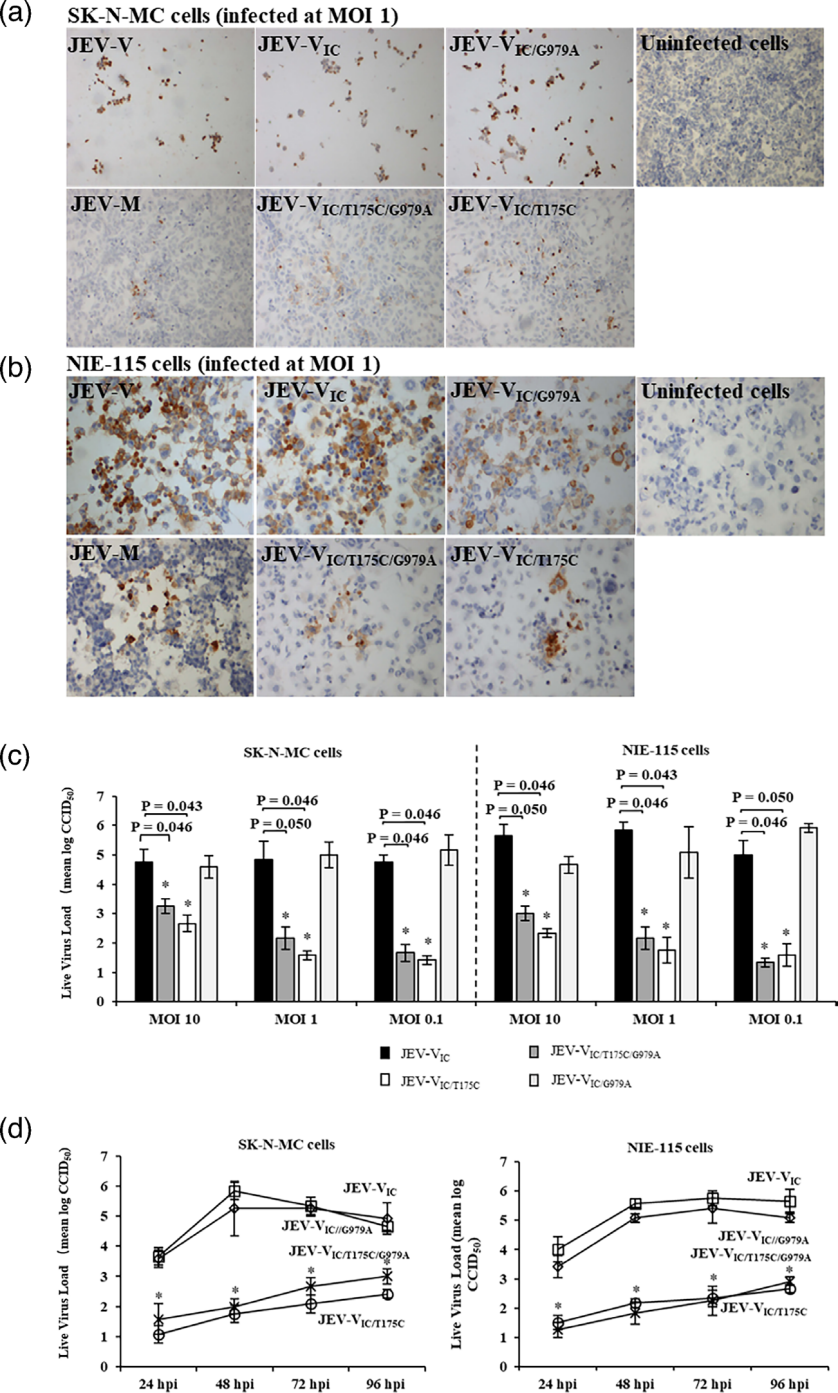
(**a**) In SK-N-MC cells infected at an MOI of 1 with JEV-V, JEV-V_IC_ and JEV-V_IC/G979A_, viral antigens were detected in the majority of remaining undetached cells. In contrast, in SK-N-MC infected with JEV-M, JEV-V_IC/T175C/G979A_ and JEV-V_IC/T175C_, only focal viral antigens were detected. Viral antigens were absent from uninfected cells. (**b**) In NIE-115 cells, infected at an MOI of 1 with JEV-V, JEV-V_IC_ and JEV-V_IC/G979A_, the majority of remaining undetached cells were viral antigen-positive. Viral antigens were absent from uninfected cells. Similar to SK-N-MC, N1E115 cells infected with JEV-M, JEV-V_IC/T175C/G979A_ and JEV-V_IC/T175C_, only focal viral antigens were detected. Viral antigens were absent from uninfected cells. (**c**) At MOIs of 10, 1 and 0.1, JEV-V_IC_ and JEV-V_IC/G979A_ clearly showed significant (*P*≤0.05) increased growth compared to JEV-V_IC/T175C/G979A_ and JEV-V_IC/T175C_ at 120 hpi. (**d**) At the MOI of 10, JEV-V_IC_, JEV-V_IC/T175C/G979A_, JEV-V_IC/T175C_ and JEV-V_IC/G979A_ growth kinetics (one-step growth curves) in SK-N-MC and NIE-115 cells showed viral titres in JEV-V_IC_- and JEV-V_IC/G979A_-infected cells to be significantly higher (*P*≤0.05) than JEV-V_IC/T175C/G979A_ and JEV-V_IC/T175C_ at all time points. JEV-V_IC_ and JEV-V_IC/G979A_ replication peaked at 48 hpi and 72 hpi, respectively, in SK-N-MC and NIE-115 and decreased gradually thereafter, while JEV-V_IC/T175C/G979A_ and JEV-V_IC/T175C_ replication gradually increased over time.

Similar to WT JEV-M, there was minimal or no CPE in SK-N-MC and NIE-115 cells infected with JEV-V_IC/T175C/G979A_ and JEV-V_IC/T175C_, clones that have the T175C mutation. Conversely, similar to WT JEV-V, progressive CPE and cell detachment were observed in JEV-V_IC_- and JEV-V_IC/G979A_-infected cells. Furthermore, at 120 hpi, only focal viral antigens were detected in JEV-V_IC/T175C/G979A_- and JEV-V_IC/T175C_-infected cells, whereas the majority of JEV-V_IC_- and JEV-V_IC/G979A_-infected cells were viral antigen-positive (Fig. 4a, b). Moreover, similar to JEV-M, significantly lower post-infection JEV-V_IC/T175C/G979A_ and JEV-V_IC/T175C_ viral titres were observed compared to JEV-V, JEV-V_IC_ and JEV-V_IC/G979A_ titres (*P*≤0.05) (Fig. 4c).

JEV-V_IC/T175C/G979A_ and JEV-V_IC/T175C_ displayed similar growth kinetics as WT JEV-M in both SK-N-MC and NIE-115 cells. Gradual increases in viral titres from 24 to 96 hpi, with titre values ranging from mean log 0.5 to 2.5 CCID_50_, were observed, but overall, JEV-V_IC/T175C/G979A_ and JEV-V_IC/T175C_ titres were significantly lower than JEV-V_IC_ and JEV-V_IC/G979A_ (*P*≤0.05). As expected, JEV-V_IC_ and JEV-V_IC/G979A_ showed similar growth kinetics to WT JEV-V (Fig. 4d).

Overall, in terms of live viral infection, JEV-V_T175C/G979A_ and JEV-V_IC/T175C_ showed closely similar *in vitro* phenotype to WT JEV-M, while JEV-V_IC_ and JEV-V_IC/G979A_ showed close similarities to WT JEV-V.

### Infectious clone with T175C mutation showed attenuated neurovirulence in the mouse model

**Fig. 5.**
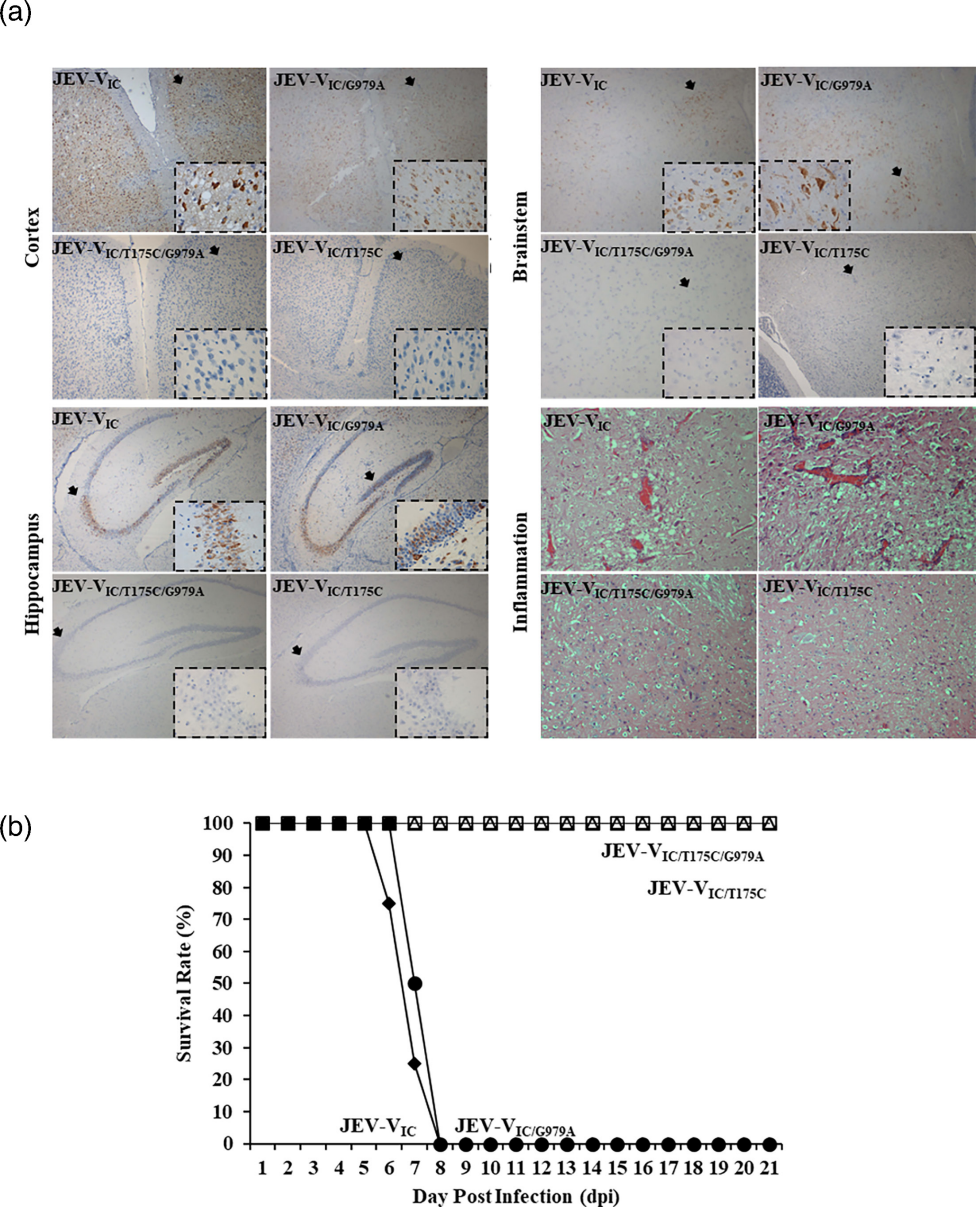
(**a**) Viral antigen and inflammation in JEV-V_IC_ and JEV-V_IC_ mutants infected mice. Viral antigen positivity and distribution in comparable and representative sections of the cerebral cortex, hippocampus and brainstem in JEV-V_IC_ and JEV-V_IC/G979A_, but not in JEV-V_IC/T175C/G979A_- and JEV-V_IC/T175C_-infected mice (original magnification: 4× objective; inset: 40× objective). The higher magnification images in the insets, taken from the areas indicated by arrows, either showed neuronal cell body cytoplasmic localization of viral antigens or their absence. Inflammation was detected in the brainstem of JEV-V_IC_ and JEV-V_IC/G979A_, but not in JEV-M-, JEV-V_IC/T175C/G979A_- and JEV-V_IC/T175C_-infected mice (original magnification: 10× objective). JEV-V_IC_- and JEV-V_IC/G979A_-infected mouse tissues were obtained at ~7 dpi, while JEV-M-, JEV-V_IC/T175C/G979A_- and JEV-V_IC/T175C_-infected animal tissues were collected at 21 dpi. All animals were footpad-infected with the same dose of 20 µl 10^4^ CCID_50_/ml. (**b**) Survival graphs of mice footpad-infected with JEV-V_IC_-, JEV-V_IC/G979A_-, JEV-V_IC/T175C/G979A_- and JEV-V_IC/T175C_.

Similar to the animals infected with 10^4^ CCID_50_/ml JEV-M, the animal groups infected with JEV-V_IC/T175C/G979A_ (*n*=4), JEV-V_IC/T175C_ (*n*=4), all survived with no signs of infection and were euthanized on 21 dpi. No histopathological lesions and viral antigens were detected in the CNS tissues (Fig. 5a).

Similar to the animals infected with JEV-V, all mice infected with JEV-V_IC_ (*n*=4) and JEV-V_IC/G979A_ (*n*=4) developed severe signs of infection or became moribund at mean 7±0.82 dpi and mean 7.5±0.58 dpi, with signs commencing at mean 6.25±0.5 dpi and 6±0.82 dpi, respectively (Fig. 5b). Neuroinflammation (Fig. 5a) and viral antigen-positive neurons were extensively detected in the cerebral cortex, hippocampus, thalamus, hypothalamus, brainstem and spinal cord as in WT JEV-V (Fig. 5). On the other hand, all uninfected control animals and JEV-V_IC/T175C/G979A_ (*n*=4), JEV-V_IC/T175C_ (*n*=4), appeared healthy throughout the experimental period, and CNS inflammation and viral antigens were absent.

## Discussion

Like all RNA viruses, orthoflaviviruses can exist as biological clones comprising a complex mixture of virions with closely related but non-identical genomes that undergo continuous mutations due to competitive selection and cooperation between clones [7,8]. The genomic diversity in biological clones is well known to confer different phenotypic characteristics that include alterations in cell tropism, virulence and host range, resistance to antiviral agents and host immune responses [7,9].

In this study, we have purified 2 JEV biological clones from a clinical isolate from the brain of a fatal JE case. In one variant (JEV-M), attenuation of neurovirulence was confirmed by *in vitro* neurovirulence testing using two neuronal cell lines (SK-N-MC and NIE-115), consistently showing less CPE and reduced growth kinetics. JEV-M attenuation was confirmed in a footpad-inoculation JE mouse model. At viral doses higher than 10^4^ CCID_50_/ml, the onset of signs of infection and death (25–50%) was dose dependent and also appeared later than the neurovirulent JEV-V. At a dose of 10^4^ CCID_50_/ml, all JEV-M-infected animals survived, while all JEV-V-infected animals succumbed to the infection with evidence of extensive CNS infection.

Two point mutations in the *E* gene and one mutation each in the NS5 gene and 3′-UTR were identified in JEV-M that were not found in the neurovirulent JEV-V. The Orthoflavivirus *E* gene has been widely associated as an important neurovirulent determinant [25,27]. Hence, in this study, we focused on two *E* gene mutations. The first, T175C, caused a tyrosine to histidine amino acid (Y59H) change in the E protein domain II, resulting in a predicted secondary structure change from *α*-helices to *β*-strands between residues 59 to 62. Our *in vitro* and *in vivo* IC data confirmed that this point mutation attenuated the neurovirulence of JEV-M, strongly suggesting that the nucleotide thymine at position 175/amino acid tyrosine at position 59 is an important neurovirulence determinant. As expected, the other *E* gene mutation, G979A, which resulted in an A327T amino acid substitution in domain III, without any predicted secondary structure change, did not seem to impact neurovirulence on its own.

For the replication cycle to start, viral attachment/binding via the E protein to the host cell surface, and subsequent viral internalization by endocytosis, has to occur. Once internalized, the E protein fuses with the endosomal membrane, allowing release of the viral genome to initiate the replication process in the cytoplasm [28,33]. We postulate that the Y59H substitution in JEV-M may have altered one or more steps involved in attachment, entry and/or release of viral genome into the cytoplasm. However, our virus-binding assay demonstrated that JEV-M retains comparable binding efficiency to neuronal cells as JEV-V, suggesting that the mutation does not impair viral attachment. Therefore, it is more likely that the attenuation observed in JEV-M is due to a disruption in post-attachment events, such as viral entry and endosomal membrane fusion. This hypothesis warrants further investigation.

The current view holds that the orthoflaviviral E protein, which comprises three main ectodomains (domains I, II and III), functions cohesively in driving this two-step viral entry process, with domain II functioning exclusively to facilitate the endosomal fusion step [31,32, 34,38]. Tyrosine in position 59 (Y59) in the *E* gene is highly conserved among representative JEV strains, including Nakayama, FU, SA14 and the vaccine strain SA14-14-2. This residue is also conserved in other orthoflaviviruses such as the Zika virus and the West Nile virus (Fig. S1). Although a leucine is present at the corresponding position in certain dengue serotypes, this variation appears to reflect natural polymorphism. To date, known mutation sites in the dengue *E* gene associated with altered viral phenotypes do not include this position [39]. While our analysis was not exhaustive, the inclusion of representative strains across the orthoflavivirus genus supports the view that Y59 is a highly conserved residue that likely plays a critical structural or functional role. Its substitution in JEV-M appears to be a rare event of potential significance. Nevertheless, the precise functional consequence of the Y59H substitution remains to be elucidated.

In previous studies, using cell lines and mouse models, *E* gene mutations/amino acid substitutions were found to attenuate viral neurovirulence and/or neuroinvasion properties in intracerebrally and intraperitoneally inoculated mice [25,27]. The amino acid changes include glutamic acid to lysine at positions 49, 138 and 306, aspartic acid to glycine at positions 389 and 390, glycine to glutamic acid at position 306, arginine to glutamic acid at position 85, arginine to serine at position 331 and arginine to methionine at position 387. The arginine to glutamic acid (position 85) is located in domain II, while the others are located on domains I and III of the E protein.

As far as we are aware, the domain II, tyrosine to histidine amino acid (Y59H) change reported herein is novel, and its relationship with neurovirulence has not been demonstrated before. Its identification could contribute to our understanding and future development of live-attenuated JEV vaccines. However, it should be noted that this amino acid substitution resulted in partial attenuation in a mouse model, which was less obvious with higher viral doses.

The impact of the Y59H mutation in the *E* gene on JEV transmission in mosquitoes remains uncertain, as to our knowledge, no studies have directly linked this specific substitution to enhanced replication or transmission in mosquito vectors. However, studies on JEV genotype I (GI) vs. genotype III (GIII) have identified non-structural protein mutations, specifically NS2B-L99V, NS3-S78A and NS3-D177E, that significantly enhance GI virus replication in amplifying hosts such as pigs and poultry, leading to higher viremia and potentially more efficient transmission in the pig/avian-mosquito cycle [40]. In another orthoflavivirus, dengue virus, an *E* gene mutation (K122E) has been shown to enhance mosquitoes’ midgut infection, thereby potentially increasing transmission efficiency [41]. These findings suggest a potential role for Y59H in modulating JEV transmission, which warrants further investigation.

Although derived from a rare brain isolate, it should be noted that the CNS138/9 viral stock and the isolated biological clones had been cell-culture passaged four times under laboratory conditions, which may, by itself, introduce mutations into the viral genome. Comparing our sequences with an earlier isolate, CNS138-11, which had been previously deposited in GenBank (GenBank accession no.: AY184213) [42], we found the *E* gene revealed three nucleotide differences at positions 175, 731 and 979, corresponding to amino acid positions 59, 244 and 327, respectively, between CNS138-11, JEV-V and JEV-M. These sequence differences could reflect mutations introduced during cell culture passaging, particularly given the quasispecies nature of RNA viruses, such as JEV. However, it is equally plausible that these differences represent pre-existing variants within the original clinical isolate. Unfortunately, the original unpassaged brain tissue from which CNS138/9 was derived is no longer available for direct sequencing to investigate this further.

With respect to the two mutations found on NS5 and 3′-UTR, previous literature has reported that the NS5 gene encodes the RNA-dependent RNA polymerase, which facilitates the viral replication process, while the 3′-UTR is important in the replication and translation process [43,44]. Our ICs that comprise the JEV-V backbone, JEV-M-derived *E* gene mutation(s), but without the NS5 and 3′-UTR mutations, showed similar replication with JEV-M, suggesting that the E protein Y59H mutation is probably the more significant neurovirulent determinant.

## Supplementary material

10.1099/jgv.0.002137 Supplementary Material 1.

## Abbreviations

CNS: central nervous system
CPE: cytopathic effects
dpi: days post-infection
*E*: envelope gene
FBS: fetal bovine serum
HDV: hepatitis D virus
hpi: hours post-infection
IC: infectious clones
ICC: immunocytochemistry
IHC: immunohistochemistry
JE: Japanese encephalitis
JEV: Japanese encephalitis virus
MOI: multiplicity of infection
RT: room temperature
TBVE: tick-borne encephalitis virus
WT: wild type
