# Supplementary material for: A Japanese encephalitis virus biological clone with an E gene point mutation exhibits in vitro and in vivo attenuation of neurovirulence
Source: J Gen Virol. 2025 Sep 1;106(9):002137. doi: 10.1099/jgv.0.002137 (PMC12401461; doi:10.1099/jgv.0.002137)
Supplement: Supplementary Material 1. [file jgv-106-02137-s001.pdf]

Supplementary Table 1: Primers for qPCR, whole genome sequencing and infectious clones

Primers for qPCR

Primer sequence (5’-3’)	Orientation
AGC AGA TTA ACC ACC ATT GG[ǂ]	Sense
CAC CAC CAC GCT GAG GTC CA[ǂ]	Antisense

Primers for JEV whole genome sequencing

Primer sequence (5’-3’)	Genome position	Orientation
AGAAGTTTATCTGCGTGAAC*	1-20	Sense
ACGTCATAGTCATCCCCACC[ϒ]	538-557	Sense
AGAAGGAGATAGCTGTTTGACAAT*	1052-1075	Sense
ACACTGGACTGTGAGCCAAG	1536- 1555	Sense
GCTCCAACTCAAAAGTGCTGG	2071-2091	Sense
CTGGAGCACCAAATGTGGGAA	2664-2784	Sense
ACACACCCTTTGGGGAGATG	3161- 3180	Sense
GGTATGTGGTGCTAGTCGCT*	3700-3719	Sense
CTATTGTTCCCGCCGCTTTTG	4555-4575	Sense
TTCTTGGCACGTATCAGGC	4696-4714	Sense
GACAGATGACTGTATTGGACCTG	5167-5189	Sense
GTGCCTCCAGAGAGCAGGG	5729-5747	Sense
CTACAAGGTGGCGTCCAATG*	6251-6270	Sense
TGCTGCTGATGGTGGTTCTC	6799-6818	Sense
AAGAACGGCGGCTGGTATAA	7328-7347	Sense
CAAAACTCCGTTGGCTCGTG	7855-7874	Sense
CCCTGTCCCGAAACTCCAAT	8305-8324	Sense
AAAAAGACCCCGCTTGTGC*	8846-8864	Sense
TCGCCCGAGCCATAATTGAA	9373-9392	Sense
ATCTCCCCAGGAGCTGGATG	9909-9928	Sense
GACAGGGTCATCTAGTGTGAT	10380-10400	Sense
AGGATGCAATAGACGAGGTG	10710-10729	Sense
GATTGCCCGGACCCAACATC	574-593	Antisense
CATCCATTTCCCCACCCAC*	1273-1291	Antisense
GTTCTCTGTTTCTCCACGCC	1670-1689	Antisense
TGAGCTCCCTTCAAAGTCGT	2202-2221	Antisense
GGATAGGCGTTTAGGGGCTG[ϒ]	2773-2792	Antisense
CCTCTTGCCACAATCCTCTGT	3339-3359	Antisense
TGATTTGTCCATTTCGCGCT*	3819-3838	Antisense
ACACTGCCATAAGACCTGCC	4319-4338	Antisense
ACGGGCCTCCATAGCTTA	4837-4854	Antisense

GGGTGTAGGCTTCTGGGACT	5129-5148	Antisense
TCTGCACTGCTGAAGTTTGA	5336-5355	Antisense
ACTCTTTCTGCAATCAATGACCCTA	5870-5894	Antisense
CTTGCATCAAGCCATCTCGG*	6387-6406	Antisense
CGTCTTTCCGCCAAACATGC	6955-6974	Antisense
TAGGATTGACGAGGAACGCTG	7462-7482	Antisense
GCCCCACCTTTCGTGTATCC	7980-7999	Antisense
TCCTCGCAGATGGTTTTCCC*	8998-9017	Antisense
TCAATCCATACCCTGTTCCAG	10127-10147	Antisense
GACCTACTTCCGAGACGGTTC	10555-10575	Antisense
AGATCCTGTGTTCTTCCTCA*	10945-10964	Antisense
GCTCATCACTACCCTCTTCACT**		Antisense
GACTCGAGTCGACATCG**		Adapter
GACTCGAGTCGACATCGATTTTTTTTTTTTTTTTT**		Adapter

Primers for the generation of infectious clones (JEV-V$_{IC}$)

Primer	Primer sequence (5'-3')	Orientation
Not-T7-JE-F	AAAAAAGCGGCCGCTAATACGACTCACTATAGGAG AAGTTTATCTGTGTGAAC	Sense
13R	CGTCTTTCCGCCAAACATGC	Antisense
24F	CTATTGTTCCCGCCGCTTTTG	Sense
HDV-10964-R	AGGCTGGGACCATGCCGGCCAGATCCTGTGTTCTTC CTCACC	Antisense
HDV-F	GGTGGTGAGGAAGAACACAGGATCTGGCCGGCATG GTCCCAGCCT	Sense
HDV-R-SWAI	CTTGCTCTAGATTTAAATAGG	Antisense
23 R	CCTCTTGCCACAATCCTCTGT	Antisense
fragment1 mt1-R#	GACTGAAGCGTGGTAGCAGT*G*ACTTCTGACCTCAGC AAGTT	Antisense
fragment2 mt1-F#	CCAACTTGCTGAGGTCAGAAGT*C*ACTGCTACCACGC TTCAGTCAC	Sense
fragment1 mt2- R#	TCCATCACTCCCAGAATACG*T*AAGCTCAATGACGAC TGTTC	Antisense
fragment2 mt2- F#	TGGAACAGTCGTCATTGAGCTT*A*CGTATTCTGGGAG TGATGGACC	Sense

* PCR primers for amplification of PCR products for Sanger sequencing

** nucleotide primers used in 5' and 3' RACE to obtain the 5' and 3' terminal of the virus genome

ϒ PCR primers for partial M, full E and partial NS1 gene amplification and sequencing,

mutated nucleotides were underlined and italicized.

Supplementary Figure 1: Amino acid at position 59 of the E protein across representative orthoflaviviruses

	Species/Abbrv	Gr	E gene position 59
JEV	1. AY184213.1_Japanese_encephalitis_virus_strain_CNS138-11		Y
JEV	2. PV210294.1_Japanese_encephalitis_virus_isolate_CNS138/9_JEV-V		Y
JEV	3. PV210295.1_Japanese_encephalitis_virus_isolate_CNS138/9_JEV-M		H
JEV	4. EF571853.1_Japanese_encephalitis_virus_strain_Nakayama		Y
JEV	5. AF217620.1_Japanese_encephalitis_virus_FU_strain		Y
JEV	6. U44964.1_Japanese_encephalitis_virus_JaGAr01		Y
JEV	7. KU323483.1_Japanese_encephalitis_virus_strain_SA14		Y
JEV	8. MK585066.1_Japanese_encephalitis_virus_strain_SA14-14-2		Y
DENV	9. KM204119.1_Dengue_virus_1_strain_Hawaii		L
DENV	10. KU725663.1_Dengue_virus_type_2_strain_16681		Y
DENV	11. KM204118.1_Dengue_virus_2_strain_New_Guinea_C		Y
DENV	12. KU050695.1_Dengue_virus_3_		L
DENV	13. AY947539.1_Dengue_virus_type_4_strain_H241		Y
ZIKV	14. MW143022.1_Zika_virus_strain_MR766		Y
ZIKV	15. MK713748.1_Zika_virus_isolate_PRVABC59		Y
WNV	16. MZ605381.2_West_Nile_virus_isolate_NY99		Y
WNV	17. AF260968.1_West_Nile_virus_strain_Eg101		Y
WNV	18. NC_001563.2_West_Nile_virus_lineage_2		Y

Tyrosine (Y) is conserved in JEV, ZIKV, and WNV, while leucine (L) is observed in some DENV strains. Histidine (H) denotes the mutant JEV-M variant.
